# Coping With Water Limitation: Hormones That Modify Plant Root Xylem Development

**DOI:** 10.3389/fpls.2020.00570

**Published:** 2020-05-15

**Authors:** Prashanth Ramachandran, Frauke Augstein, Van Nguyen, Annelie Carlsbecker

**Affiliations:** Department of Organismal Biology, Physiological Botany, Evolutionary Biology Centre and Linnean Centre for Plant Biology, Uppsala University, Uppsala, Sweden

**Keywords:** Arabidopsis, drought, root, development, xylem

## Abstract

Periods of drought, that threaten crop production, are expected to become more prominent in large parts of the world, making it necessary to explore all aspects of plant growth and development, to breed, modify and select crops adapted to such conditions. One such aspect is the xylem, where influencing the size and number of the water-transporting xylem vessels, may impact on hydraulic conductance and drought tolerance. Here, we focus on how plants adjust their root xylem as a response to reduced water availability. While xylem response has been observed in a wide array of species, most of our knowledge on the molecular mechanisms underlying xylem plasticity comes from studies on the model plant *Arabidopsis thaliana*. When grown under water limiting conditions, Arabidopsis rapidly adjusts its development to produce more xylem strands with altered identity in an abscisic acid (ABA) dependent manner. Other hormones such as auxin and cytokinin are essential for vascular patterning and differentiation. Their balance can be perturbed by stress, as evidenced by the effects of enhanced jasmonic acid signaling, which results in similar xylem developmental alterations as enhanced ABA signaling. Furthermore, brassinosteroids and other signaling molecules involved in drought tolerance can also impact xylem development. Hence, a multitude of signals affect root xylem properties and, potentially, influence survival under water limiting conditions. Here, we review the likely entangled signals that govern root vascular development, and discuss the importance of taking root anatomical traits into account when breeding crops for enhanced resilience toward changes in water availability.

## Root Xylem Characteristics Are Influenced by Changes in Water Availability

Agricultural drought refers to conditions of insufficient water availability rendering conditions unsuitable for plant growth (Wilhite and Glantz, 2009). Understanding mechanisms of plant response to water limitation can help in the breeding of crops with enhanced survival under such conditions. For long, focus has been put on above ground traits or root system architectural properties, but recently more attention has been given to how anatomical parameters and, in particular, xylem structures of the roots influence water transport and drought resilience. The tracheary elements of the xylem form hollow vessels or tracheids that are structurally reinforced with lignified secondary cell walls (SCW), providing the ability to withstand the strong negative pressure generated by the transpiration pull and promote bulk water movement from the roots to the shoot. The geometrical and physical properties of the tracheary elements influence water transport capacity and research in a wide array of species suggests that xylem traits are important for the ability of plants to withstand periods of reduced water availability (Lucas et al., 2013). The importance of root xylem characteristics for drought tolerance was recently underscored by a study identifying *Arabidopsis thaliana* (Arabidopsis) ecotypes with enhanced root hydraulic conductance (Tang et al., 2018). Through genome wide association studies this trait was linked to XYLEM NAC DOMAIN1 (XND1) (Tang et al., 2018), a well-known negative regulator of xylem differentiation (Zhao et al., 2008, 2017). The *xnd1* loss of function mutants in the Col-0 ecotype had increased root xylem area, and higher aquaporin activity, resulting in enhanced hydraulic conductance compared to wild type, and these plants also displayed enhanced drought tolerance on soil (Tang et al., 2018). Similar root anatomical traits were associated with enhanced hydraulic conductance, drought tolerance and increased yield in field grown soy bean (*Glycine max)* plants (Prince et al., 2017). Interestingly, wheat varieties bred to instead possess smaller xylem diameter displayed higher grain yield during drier growth periods because of improved use of subsoil water (Richards and Passioura, 1989). In line with this, drought exposed rice may respond with formation of smaller xylem diameter (Henry et al., 2012). This strategy is similar to what is observed in drought stressed poplar (*Populus nigra* L. × *Prunus maximowiczii*) trees, which adjust their xylem development to produce thinner but more xylem vessels in their wood (Arend and Fromm, 2007). Thinner xylem vessels increase resistance but reduce risk of embolisms, which occurs under water limiting conditions (Lucas et al., 2013). Thus, different species may benefit from different strategies, but the occurrence of xylem modifications under drought in different species grown under both lab and field conditions suggests these to be important adaptive responses to water limitation. Hence, the molecular mechanisms underlying these responses are potentially important targets for crop breeding programs. Here, we discuss a number of hormones and small molecules known, primarily from studies in Arabidopsis, to affect root xylem patterning and differentiation and how the current knowledge can be employed to optimize plant behavior under normal and drought conditions.

## ABA Regulates Xylem Development Via miRNA165

**FIGURE 1:**
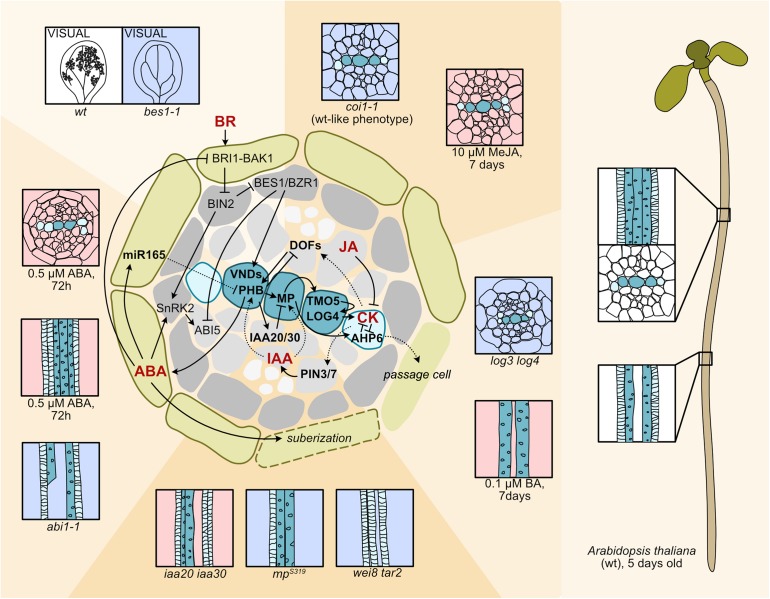
Hormone circuits controlling root xylem development. In the Arabidopsis seedling root, to the right, spiral-walled protoxylem vessels (light blue-green) differentiate first followed by the pitted metaxylem vessels (dark blue-green). To the left a cartoon depicting a cross section focusing on the stele surrounded by the endodermis. Cell types are as indicated: endodermis (green), pericycle (dark gray), procambium (light gray), protoxylem (light blue-green), metaxylem (dark blue-green). Signaling pathways affecting xylem patterning and differentiation are shown on top of the cross section. Hormones are in bold red letters. Arrows indicate activation, bars inhibition. Dashed arrows indicate movement. Phenotypic consequences of hormone treatments or biosynthesis/signaling perturbations for selected experiments are displayed around the cross section. Decreased hormone levels/signaling (light blue background), enhanced levels/signaling (light red background). A PIN3/7 mediated lateral transport focuses auxin (IAA) to a central axis within the stele (Bishopp et al., 2011). Here, auxin-activated MP induces *TMO5* that activates *LOG3* and *LOG4* resulting in CK biosynthesis (De Rybel et al., 2014; Ohashi-Ito et al., 2014). MP also activates AHP6 which inhibits CK signaling (Bishopp et al., 2011). CK moves to the procambium and activates *PIN3* and *7*, and DOF TFs (Bishopp et al., 2011; Miyashima et al., 2019; Smet et al., 2019). MP is required for xylem formation, as the weak *mp^S319^* mutant has discontinuous protoxylem and mutants defective in the MP repressors IAA20 and IAA30 result in additional protoxylem (Müller et al., 2016). The auxin biosynthesis mutant *wei8 tar2* lacks metaxylem because of reduced HD-ZIP III expression (Ursache et al., 2014). The cytokinin biosynthesis mutant *log3 log4* has extra protoxylem and a wider xylem axis (De Rybel et al., 2014; Ohashi-Ito et al., 2014), whereas treatment with the synthetic CK, 6-benzylaminopurine (BA) results in loss of protoxylem due to *AHP6* suppression (Argyros et al., 2008; Bishopp et al., 2011). JA activates *AHP6* expression and suppresses *PIN7* expression (Jang et al., 2017, 2019). Methyl-JA treatment results in extra protoxylem and a wider xylem axis, but mutation in the JA receptor COI does not affect xylem development (Jang et al., 2017). ABI1 mediated ABA signaling in endodermis induces miR165 and miR166, which move into the stele to restrict HD-ZIP III mRNA, exemplified with *PHABULOSA* (*PHB*) (Ramachandran et al., 2018; Bloch et al., 2019). ABA treatment results in protoxylem in place of metaxylem and extra protoxylem, while ABA signaling and biosynthesis mutants display xylem breaks (Ramachandran et al., 2018). Endodermal ABA signaling enhances suberization (Barberon et al., 2016). Mobile AHP6 represses suberization resulting in passage cells for water and nutrient uptake (Andersen et al., 2018). ABA signaling components interact with BR signaling resulting in antagonistic control of downstream targets. ABA signaling activates ABI5, while *ABI5* expression is repressed by BES1/BZR1 via BRI1-BAK1 receptor and BIN2 GSK3-mediated BR signaling, and BIN2 interferes with ABA signaling by activating SnRK2 kinases (Planas-Riverola et al., 2019). BR activates VND TFs that induce xylem differentiation. In the *in vitro* vascular cell induction system VISUAL, formation of ectopic xylem is inhibited in the BR signaling mutant *bes1−1* (Saito et al., 2018).

Under conditions of reduced water availability, *in vitro*-grown Arabidopsis responds with reduced root growth and suppressed lateral root development (Rowe et al., 2016). Recently, it was found that this also causes major changes to the root’s internal anatomy (Jang and Choi, 2018; Ramachandran et al., 2018; Bloch et al., 2019). Normally, the Arabidopsis root stele has a diarch anatomy: a xylem axis traverses the stele with one strand of protoxylem with annular or spiral SCW at either end of the axis and metaxylem with pitted SCW in the center (Figure 1). When water availability is reduced, additional protoxylem strands form, both to widen the axis and to shift the identity of the xylem strands within the axis such that protoxylem develops in metaxylem positions (Jang and Choi, 2018; Ramachandran et al., 2018; Bloch et al., 2019). Identity changes were observed also under exogenous treatment with ABA, a well-known mediator of abiotic stress (Zhu, 2016), even below root growth-inhibiting concentrations (Figure 1). These phenotypic alterations were strongly attenuated when ABA signaling was compromised, suggesting that they are ABA mediated. Strikingly, inhibition of ABA signaling in the endodermis cell-layer, surrounding the stele, was sufficient to partially suppress xylem identity changes, indicating that ABA acts via a non-cell-autonomous signal (Ramachandran et al., 2018; Bloch et al., 2019). The microRNAs, microRNA165 (miR165) and miR166, are well-known signals moving from endodermis into the stele to determine xylem cell identity (Carlsbecker et al., 2010; Miyashima et al., 2011). These miRNAs are produced in the endodermis but move into the stele to target mRNAs of class III homeodomain leucine-zipper (HD-ZIP III) transcription factors (TFs). The lower levels of HD-ZIP III TFs in the periphery compared to the central stele determine protoxylem and metaxylem identity in the peripheral and central positions of the xylem axis, respectively (Carlsbecker et al., 2010; Miyashima et al., 2011). Hence, upon elevated miR165 levels or in HD-ZIP III loss-of-function mutants, protoxylem forms in the place of metaxylem, conspicuously similar to the phenotype observed under limited water availability or ABA treatments. Indeed, under water-limiting conditions miR165 production in the endodermis is enhanced and, consequently, HD-ZIP III TF levels reduced, explaining the observed shift in xylem cell identity (Ramachandran et al., 2018; Bloch et al., 2019). Intriguingly, if miR165/166 levels instead are strongly reduced throughout the Arabidopsis plant, by the use of an artificial miRNA-target that sequesters miR165/166 (STTM165/166), it results in elevated expression of ABA-related genes and enhanced drought tolerance (Yan et al., 2016). Similar approach conferred drought tolerance also in rice, however in rice miR166 is expressed only in the shoot and consequently only leaf and stem xylem number were affected (Zhang et al., 2018). Since the HD-ZIP III TFs can influence leaf morphology as well as root xylem development, further studies are needed to investigate if these factors could be differentially regulated in roots and shoot upon water stress, and how they may contribute to ABA homeostasis.

## Auxin-Cytokinin Interplay Patterns the Root Vasculature

Under normal development, research on Arabidopsis embryos and roots has shown that auxin plays a key role in establishing vascular patterns where xylem and phloem are separated by intervening procambium (Figure 1; Bishopp et al., 2011). Central for this is the TF AUXIN RESPONSE FACTOR5 (ARF5)/MONOPTEROS (MP) (Berleth and Jürgens, 1993; Bishopp et al., 2011). High levels of auxin, primarily within the xylem precursors, activate *MP*, which in turn induces *TARGET OF MONOPTEROS5* (*TMO5*) (Schlereth et al., 2010). TMO5 in complex with LONESOME HIGHWAY (LHW), controls procambial periclinal cell divisions (Ohashi-Ito et al., 2013), by promoting cytokinin (CK) biosynthesis via the activation of *LONELY GUY3* (*LOG3*) and *LOG4* (De Rybel et al., 2014; Ohashi-Ito et al., 2014). Although CK is synthesized within the xylem domain, CK response is low here (Bishopp et al., 2011). Instead, CK is sensed in the neighboring procambial cells, where it activates several DNA-binding one finger (DOF) TFs to promote procambial periclinal cell divisions (Miyashima et al., 2019; Smet et al., 2019). CK also promotes the expression of auxin efflux carriers PIN3 and PIN7, which move auxin laterally into the xylem domain (Bishopp et al., 2011). Auxin, in the protoxylem positions, induces *HISTIDINE PHOSPHOTRANSFER PROTEIN6 (AHP6)* (Bishopp et al., 2011), a negative regulator of CK signaling (Mähönen et al., 2006), partially explaining the reduced CK response and limited periclinal cell divisions within the xylem axis. Within the central xylem axis, auxin biosynthesis promotes HD-ZIP III transcription (Ursache et al., 2014), and it is possible that these factors contribute to the suppression of CK signaling, as they can inhibit B-type response regulators (B-ARRs) under conditions of high CK levels (Sebastian et al., 2015). Modeling approaches have shown that the above described interactions are sufficient to generate *de novo* patterning, replicating both a diarch and more complex anatomical patterns that are seen in other plant species, primarily depending on the size of the stele (Mellor et al., 2017, 2019). The patterning factors are further intertwined, as the HD-ZIP III TFs both interfere with auxin signaling (Müller et al., 2016), and suppress expression of cytokinin induced DOF TFs, while certain DOF TFs move from the phloem to positively influence HD-ZIP III expression in intervening procambial cells (Miyashima et al., 2019). Hence, it is conceivable that, similar to ABA’s influence on miR165/HD-ZIP III TFs, this complex network is targeted at multiple points by abiotic signals to alter xylem development. It remains to be examined if the formation of extra xylem strands, widening the xylem axis, observed under water limiting conditions, is the effect of ABA impinging on the delicate auxin-cytokinin balance that normally demarcates domains of low and high periclinal division activity. Multiple examples where abiotic stresses, and ABA specifically, intersect with and affect auxin and cytokinin can be found in other contexts for example in the regulation of seed germination, cell elongation and root growth (Verslues, 2016; Bielach et al., 2017; Huang et al., 2018). Such an intersection may therefore be anticipated also in the regulation of vascular patterning.

## Abiotic Stress Affects Root Xylem Differentiation to Influence Drought Tolerance

The xylem precursor cells, patterned and specified by the auxin-cytokinin/HD-ZIP III regulatory networks, differentiate into functional xylem vessels through a differentiation program involving programmed cell death and SCW deposition (reviewed by Furuta et al., 2014). Apart from XND1, TFs of another NAC subfamily, VASCULAR NAC DOMAIN (VND), are master regulators of xylem differentiation, and overexpression of any of the seven VND-genes result in trans-differentiation of other cell types into tracheary element cells (Kubo et al., 2005; Endo et al., 2015). A hierarchical TF network with VNDs regulating two tiers of MYB domain TFs acts directly upstream of lignin and cellulose biosynthesis genes (Taylor-Teeples et al., 2014; Turco et al., 2019). Network perturbation analysis revealed that one of the HD-ZIP III TFs, REVOLUTA is a negative regulator of lignin biosynthesis, and that the network modulates xylem development under conditions of iron deficiency or salt stress (Taylor-Teeples et al., 2014). The increase in expression of lignin biosynthesis genes under iron deficient conditions is dependent on reduction in *REV* levels, while *MYB46* and *VND7* play crucial roles in enhancing xylem differentiation during salt stress (Taylor-Teeples et al., 2014). Thus, the presence of several upstream regulators of SCW biosynthesis allows the use of specific TFs in response to different types of stresses. Interestingly, in apple, MdMYB88 and MdMYB122 were found to influence hydraulic conductivity by affecting xylem density, diameter, and the expression of SCW biosynthesis genes (Geng et al., 2018). The activation of SCW biosynthesis genes to maintain root hydraulic conductivity during drought stress was found to be through their direct regulation of *MdVND6* and *MdMYB46*, suggesting that co-option of xylem development regulators maybe be evolutionarily conserved.

Intriguingly, low levels of ABA, even under non-stressed conditions, are required for the formation of continuous xylem strands, since both ABA-biosynthesis and signaling mutants have patches along the xylem strands that are either retained in an undifferentiated procambial state or are xylem cells with defective SCW formation (Figure 1; Ramachandran et al., 2018). Suppression of ABA signaling in cell-layers external to the stele, such as in the endodermis or epidermis also resulted in similar discontinuous xylem suggesting a non-cell autonomous effect of ABA. Indeed, inhibition of ABA biosynthesis or suppression of endodermal ABA signaling reduced *MIR165A* levels and consequently elevated the expression of certain HD-ZIP III genes (Ramachandran et al., 2018). ABA is also important during secondary development as ABA biosynthesis mutants exhibit delayed xylem fiber formation (Campbell et al., 2018). Contrastingly, exogenous ABA treatment induces protoxylem differentiation closer to the root tip in Arabidopsis and tomato (Bloch et al., 2019) suggesting that in addition to interfering with xylem identity ABA promotes differentiation. Interestingly, endodermal ABA signaling acts in a similar manner to promote suberization of the endodermis (Figure 1; Barberon et al., 2016). The movement of AHP6 from protoxylem precursors and neighboring pericycle cells to the endodermis represses cytokinin signaling allowing the formation of “passage cells” lacking suberization for the entry of water and nutrients into the stele. Increase in ABA levels enhances endodermal suberization and reduces passage cell number (Andersen et al., 2018). It will be important to further explore how the differentiation programs of xylem and endodermis are intertwined and how this may influence radial conductivity of water and nutrients. Furthermore, endodermal ABA signaling can also affect lateral root development (Duan et al., 2013), hinting toward the endodermis as a hub for multiple developmental changes upon drought, from xylem patterning to root architecture.

## Brassinosteroids and Thermospermines Affect Xylem Differentiation and Impact on Abiotic Stress Tolerance

Use of Arabidopsis and *Zinnia in vitro* cell culture systems, where cells are triggered to trans-differentiate into xylem cells, have identified brassinosteroids (BR) as molecular cues that promote xylem differentiation (Yamamoto et al., 1997; Tan et al., 2019). The addition of BR or chemical inhibitors of BR signaling repressors to culture media containing auxin and cytokinin promoted xylem differentiation in a VND-dependent manner (Kondo et al., 2014; Tan et al., 2018). Although BR and ABA seem to act similarly with respect to promotion of xylem differentiation, there is substantial evidence for BR-ABA antagonism at several levels. BR and ABA responsive TFs, *BRI1 EMS SUPPRESSOR1* (*BES1*) and *RESPONSIVE TO DESICCATION* (*RD26*), respectively, share common targets but regulate them in opposing ways (Chung et al., 2014). Under normal conditions, BR signaling promotes growth in a BES1 dependent manner, however, upon exposure to stress the activation of RD26 inhibits BR mediated growth through the regulation of BES1 targets. Interestingly, while the application of BR promotes drought tolerance in a concentration dependent manner, genetic evidence indicates that loss of BR receptor function can also confer drought tolerance (reviewed by Nolan et al., 2020). Adding to the complexity, the overexpression of one of the BR receptors, BRASSINOSTEROID INSENSITIVE1 LIKE3 (BRL3) also conferred drought tolerance, without affecting growth, through the accumulation of osmoprotectant sugars in the root (Fàbregas et al., 2018). The antagonistic function of BR and ABA in growth modulation, but their similar effects in promoting xylem formation, raises the question of whether the two hormones might regulate similar sets of genes but under different conditions thus providing a frame work to regulate xylem development independent of growth inhibition. Also, other molecules need to be included into the equation: in the BR receptor mutant *bri1*, the root procambial cells differentiate into xylem, resulting in an increased number of xylem vessels in a BR independent manner. This is due to the positive effect that BRI1 exerts on phytosulfokine (PSK) signaling, and mutants defective in PSK signaling display similar ectopic xylem differentiation in procambial positions (Holzwart et al., 2018). The involvement of BRI1 in BR, ABA, and PSK signaling provides challenges to dissect the individual roles of these components in controlling xylem development and if they function together in stress mediated xylem modifications.

Another molecule with a capacity to regulate xylem differentiation is the polyamine thermospermine. This molecule represses xylem differentiation, as mutations in the thermospermine synthase gene, *ACAULIS5* (*ACL5)*, result in earlier xylem differentiation (Muñiz et al., 2008). Furthermore, ACL5 influences procambial divisions as thermospermine affects the translation of the auxin induced SUPPRESSORS OF ACAULIS51 LIKE (SACL) group of bHLH TFs. The SACL TFs are paralogs to TMO5, and compete for dimerization with LHW, thereby restricting TMO5-mediated promotion of procambial divisions (Katayama et al., 2015; Vera-Sirera et al., 2015). Interestingly, the *acl5* mutant, which has excess xylem formation, is salt sensitive while mutations in the gene encoding a thermospermine catabolizing enzyme, *POLYAMINE OXIDASE 5 (PAO5)*, or treatment with thermospermine which results in fewer xylem vessels, rendered the plant tolerant to salt stress (Shinohara et al., 2019). Thus, here fewer xylem strands correlate with an increased tolerance to salt stress, possibly by reducing the systemic spread of salt toxicity. However, *acl5* mutants displayed wildtype-like sensitivity when exposed to drought and mannitol treatments suggesting that different mechanisms are at play in mediating salt and drought stress tolerance. Interestingly, *pao5* mutants, which show elevated levels of thermospermine, spermine, and N’-acetyl spermine and have fewer xylem vessels in the root display tolerance to drought and reduced sensitivity to ABA thus indicating that the levels of these molecules can be modulated during stress to alter xylem development (Shinohara et al., 2019). A study in poplar revealed that thermospermine level established by a negative feedback regulation between ACL5, auxin and the HD-ZIP III TF ATHB8 is important for proper xylem differentiation (Milhinhos et al., 2013). Further investigations into the roles of these polyamines and how they function together with other xylem development regulators during stress will be important to understand how polyamine modulation can confer stress tolerance.

## Long Distance Signaling Components Influencing Xylem Development

To cope with environmental stressors, plants have developed an array of long-distance signaling cascades that include hydraulic, electrical, and chemical signals (Huber and Bauerle, 2016). An example of how such long-range signals can impact root xylem development comes from experiments where wounding of Arabidopsis cotyledons resulted in hydrogen peroxide accumulation in the root causing root xylem differentiation closer to the root tip (Fraudentali et al., 2018). Jasmonic acid (JA), a wound induced signal, may be one such long-range signal as JA was found to cause hydrogen peroxide accumulation and early xylem differentiation (Ghuge et al., 2015). It has been suggested that JA and CK signaling pathways have antagonistic interactions (reviewed by O’Brien and Benková, 2013) and they play similar antagonistic roles in xylem development. Exogenous application of methyl-JA for long periods caused the formation of extra xylem strands by promoting xylem differentiation of procambial cells. This xylem promoting effect of JA was accomplished by interference with the auxin/cytokinin balance within the stele, through ectopic activation of AHP6, which suppresses cytokinin response, and repression of *PIN7* expression within the procambial domain (Jang et al., 2017, 2019). Further, reduced water availability activated the expression of JA responsive genes, LIPOXYGENASE2 (*LOX2)* and JASMONATE INSENSITIVE 1 (*JAI1*/*MYC2)*, indicating that during drought stress JA signaling might be another pathway involved in xylem developmental plasticity (Jang and Choi, 2018).

A recent study identified the CLAVATA3/EMBRYO-SURROUNDING REGION-RELATED25 (CLE25) peptide to act as a mobile signal from the root to the leaves under dehydration conditions (Takahashi et al., 2018). Application of CLE25 to Arabidopsis seedlings, induced ABA biosynthesis and resulted in ABA mediated stomatal closure (Takahashi et al., 2018). The receptor BARELY ANY MERISTEM1 (BAM1) involved in CLE25 signaling, also associates with CLE9/10 to restrict xylem cell number. Mutants defective in *CLE9/10* display increased periclinal cell divisions within the xylem axis resulting in more xylem vessels (Qian et al., 2018). Interestingly, in cotyledons, CLE9 perception and signaling through a different receptor, HAESA-LIKE1, negatively affects the number of guard cells (Qian et al., 2018). Hence, the mobility of CLE peptides and their ability to control two aspects of plant development that are involved in hydraulic conductance warrants further investigation into how these peptides might coordinate drought stress responses in the root and shoot.

## What Can We Look Forward To?

Studies on Arabidopsis have revealed how different regulatory components influence root xylem developmental. Existing evidences point toward the repurposing of core developmental regulators to bring about phenotypic alterations in response to environmental perturbations. However, there are missing links on how different environmental inputs are interpreted by the plant. Recent progress in single cell sequencing technologies will help identify how the developmental trajectories of specific cell types are altered by external stimuli and find components central to phenotypic plasticity (Rodriguez-Villalon and Brady, 2019; Ryu et al., 2019; Shulse et al., 2019). The understanding of plant response to water stress requires simultaneous monitoring of various physiological characteristics, such as modifications to the xylem vessel diameter and number, properties of the cell wall such as lignification or suberization and composition of the soil-root-microbiome interface (reviewed by Lynch et al., 2014). Plant imaging platforms such as *light* sheet fluorescence microscopy and Growth and Luminescence Observatory for Roots (GLO-Roots) allow not only the analysis of root system architecture and anatomical phenes but also the visualization of gene expression patterns, enabling the simultaneous characterization of responses at physiological and molecular levels (Rellán-Álvarez et al., 2015; von Wangenheim et al., 2020). In addition, computational simulation tools such as GRANAR, which facilitate studies on the effect of different monocot root anatomies on root hydraulic conductivity (Heymans et al., 2020) or OpenSimRoot, which can be used to reconstruct root systems, in combination with hydraulic models, will aid the study of anatomical parameters that influence water transport (Postma et al., 2017). One has to bear in mind, though, that varieties that constitutively employ theoretical water saving strategies are not always best suited for real world growth regimes (Skirycz et al., 2011). Rather, the future of agriculture likely lies in the generation of “personalized crops” that are designed to suit the climate, soil properties and microbiota of a certain region. To meet such a goal, multiple approaches will be needed, including further exploration into the extent of natural variation. Interestingly, the Arabidopsis C24 ecotype has been found to be tolerant to multiple stress factors and has a unique combination of low water use and high seed biomass (Bechtold et al., 2010; Bechtold et al., 2018), thus the underlying genetics of this and similar studies on naturally occurring stress tolerant populations of a species can guide approaches in crop breeding. Alternatively, available knowledge on regulatory networks such as those described in this review can be harnessed to alter phenotypes specifically and rationally.

## Author Contributions

PR, FA, VN, and AC wrote the manuscript together. FA made the illustration, with input from the other authors.

## Conflict of Interest

The authors declare that the research was conducted in the absence of any commercial or financial relationships that could be construed as a potential conflict of interest.
